# Unlocking potentials: the impact of meropenem, meropenem-vaborbactam, and ceftazidime-avibactam in combating carbapenem-resistant *Enterobacter cloacae*

**DOI:** 10.1128/aac.00906-25

**Published:** 2025-11-19

**Authors:** Thomas Lavoie, Teresa Sierra, Katie Daffinee, Jason M. Pogue, Brahim Achour, Kerry LaPlante

**Affiliations:** 1 Infectious Diseases Research Program, Providence Veterans Affairs Medical Center 656595 , Providence, Rhode Island, USA; 2 College of Pharmacy, University of Rhode Island 4260, Kingston, Rhode Island, USA; 3 Department of Clinical Pharmacy, University of Michigan College of Pharmacy, Ann Arbor, Michigan, USA; 4 Center of Innovation- Long-term Services and Supports, Providence Veterans Affairs Medical Center 583504 , Providence, Rhode Island, USA; Providence Portland Medical Center, Portland, Oregon, USA

**Keywords:** *Enterobacterales*, *Enterobacter cloacae*, carbapenem-resistant, meropenem, meropenem-vaborbactam, ceftazidime-avibactam

## Abstract

Carbapenem-resistant *Enterobacterales* (CREs) are recognized as an urgent public threat by the Centers for Disease Control and Prevention. This study assesses the activity of humanized serum concentrations for meropenem (2 g Q8h), meropenem-vaborbactam (4 g Q8h), and ceftazidime-avibactam (2.5 g Q8h) against carbapenemase-producing (CP) (AR-1046*_bla_*_KPC-3_) and non-CP (AR-8*_bla_*_ACT-15_) *Enterobacter cloacae* isolates in a 120 h *in vitro* pharmacodynamic model. Additionally, we compared antimicrobial administration as a bolus or extended infusion for each isolate and treatment. For the CP strain, all treatments with meropenem-vaborbactam and ceftazidime-avibactam were bacteriostatic through 24 h. Extended-infusion meropenem-vaborbactam exhibited the most prolonged bacteriostatic activity and was significantly more effective than ceftazidime-avibactam at 120 h (0.92 log_10_ lower mean colony count compared to extended-infusion ceftazidime-avibactam, *P* < 0.001). For the non-CP strain, all simulated treatments demonstrated initial bactericidal activity through 24 h, except for ceftazidime-avibactam bolus administration. Meropenem and meropenem-vaborbactam, administered as bolus dosing, each maintained significantly greater bacterial colony count reductions compared to extended infusion through 120 h against non-CP CRE (*P* < 0.01). No significant differences were found between bolus and extended infusion for ceftazidime-avibactam (*P* = 0.08). Resistance development was observed following treatment with meropenem monotherapy (minimum inhibitory concentrations increasing to >32 µg/mL for the non-CP isolate with extended infusion and to >32 µg/mL for the CP isolate with bolus and extended-infusion treatments) and ceftazidime-avibactam (minimum inhibitory concentration increase from 2 to 16 μg/mL with bolus dosing against the CP isolate). Meropenem-vaborbactam exposure did not result in the emergence of antimicrobial resistance in any of the tested models.

## INTRODUCTION

Carbapenem-resistant *Enterobacterales* (CREs) are listed as urgent threats on the Centers for Disease Control and Prevention (CDC) Antimicrobial Resistance Threats report. They have become a major worldwide public health threat (1). Invasive infections due to CRE have historically been associated with high mortality rates, ranging from 30% to 40% with polymyxin-based therapies, and frequently impact the most vulnerable patient populations (2–5).

Gram-negative bacteria harbor intrinsic and acquired resistance mechanisms such as degradative enzymes, efflux pumps, and porin channel mutations. CRE typically constitutes the presence of carbapenemase enzymes (6). Nearly half (~47%) of all CREs lack carbapenemases; however, the absence of carbapenemases is less common (approximately 20% of isolates) when defining CRE as either resistance to one of meropenem or imipenem (7, 8). The most frequent organism classified as non-carbapenemase-producing (CP) CRE is the *Enterobacter* species (9). Infections caused by CRE are associated with high morbidity and mortality rates regardless of CP (10).

Appropriate antimicrobial selection for the treatment of drug-resistant *Enterobacterales* is crucial to ensure timely and effective treatment (11). Historically, carbapenems have been a reliable option for even the most serious and resistant infections (6, 12). Fortunately, their efficacy has been largely retained, with a decline in resistance being observed in many *Enterobacterales* in the United States. Antimicrobial surveillance data, however, indicate that this trend differs for *Enterobacter cloacae*, where carbapenem-resistance is becoming more prevalent (13). Current CRE treatment guidance from the Infectious Diseases Society of America (IDSA) states that treatment of non-CP CRE is currently guided by susceptibility testing results to carbapenems and the severity of infection, listing meropenem as a viable option in isolates with lower MIC values; however, preclinical and clinical evidence to support this recommendation is lacking (14). Furthermore, minimal data exist evaluating whether extended-infusion (EI) meropenem treatment alone is an effective treatment for non-CP CRE compared to the addition of β-lactamase inhibitors, such as vaborbactam to meropenem, which may modestly assist in decreasing MICs through the inhibition of enzyme activity due to serine-based class A carbapenemases (14). Therefore, *in vitro* data assessing isolates with MICs grouped around respective breakpoints can be a valuable tool to assist clinicians with clinical decision-making.

Ceftazidime-avibactam and meropenem-vaborbactam have safety and clinical data supporting their superiority over prior treatment of CRE with polymyxin-based regimens (15, 16). Data from the now antiquated CRE treatment, with colistin-based therapy, have consistently shown differences between agents with worse clinical outcomes compared to β-lactam β-lactamase inhibitors (BLBLIs) (2, 17–19). Appropriate use of these newer antimicrobials is essential to prolong their utility. Comparative data, however, evaluating the activity of ceftazidime-avibactam and meropenem-vaborbactam for the treatment of CRE infections remain limited (14). Therefore, the primary goal of this study was to assess the efficacy and propensity for resistance selection of meropenem alone, meropenem-vaborbactam, and ceftazidime-avibactam against both CP and non-CP CRE in *E. cloacae* using concentrations derived from infected patients.

## MATERIALS AND METHODS

### Bacterial strains

We examined two unique carbapenem-resistant *Enterobacter cloacae* provided by the CDC and the Food and Drug Administration Antibiotic Resistance (AR) Isolate Bank. Isolates were selected based on MIC and known resistance mutations. AR-8*_bla_*_ACT-15_ was selected to analyze the efficacy of each antimicrobial in the setting of AmpC enzymes coupled with porin mutations. AR-1046*_bla_*_KPC-3_ was chosen to assess a *Klebsiella pneumoniae* carbapenemase-producing isolate with a single porin mutation. Isolate-specific mutations and MICs can be found in Table 1.

**TABLE 1:** Isolate characteristics and baseline isolate minimum inhibitory concentrations^*a*^

Isolate	β-Lactamase(s)	Porin mutations	MIC (μg/mL)		
			Meropenem	Meropenem / vaborbactam	Ceftazidime / avibactam
AR-8^*b*^	ACT-15	Omp36, OmpC, OmpC2, OmpF2, OmpK36	2	0.5	1
AR-1046^*c*^	KPC-3, OXA-9, and TEM-1A	OmpF2	8	0.5	2

^*a*^ Clinical and Laboratory Standards Institute breakpoints used for susceptibility: meropenem ≤1, meropenem-vaborbactam ≤4/8, and ceftazidime-avibactam ≤8/4.

^*b*^ MIC shifts seen in AR-8 treatment models: extended-infusion ceftazidime-avibactam (MIC shifts from 1 to 4 μg/mL after 72 h) and extended-infusion meropenem (MIC shifts from 2 to >32 μg/mL after 120 h).

^*c*^ MIC shifts seen in AR-1046 treatment models: bolus ceftazidime-avibactam (MIC shifts from 2 to 16 μg/mL after 120 h) and extended-infusion and bolus meropenem (MIC shifts from 8 to >32 μg/mL after 24 h).

### Medium and antimicrobials

Meropenem-vaborbactam (vaborbactam powder, Melinta Therapeutics lot number LFRB7B2008), meropenem (Pfizer Inc. lot number 0002E21), and ceftazidime-avibactam (Allergan Pharmaceutical lot number 22K00378) were used in the experiments. Drug products were supplied by the Providence Veterans Affairs Medical Center Pharmacy Department, except for vaborbactam, which was supplied by Melinta Therapeutics. Antimicrobial stability was ensured in accordance with drug monographs and Trissel’s Stability of Compounded Formulations accessed via Micromedex. Cation-adjusted (calcium, 25 μg/mL, and magnesium, 12.5 μg/mL) Mueller-Hinton broth (CAMHB) was used in accordance with Clinical and Laboratory Standards Institute (CLSI) to determine susceptibility testing and for *in vitro* models (20).

### Targeted humanized serum concentrations

We tested free drug concentrations derived from population pharmacodynamic (PD) data in infected patients at steady state. While the meropenem-vaborbactam United States Prescribing Information (USPI) does provide mean pharmacokinetic (PK) data in infected patients, this population analysis is not available within the ceftazidime-avibactam USPI. Mean PK data for ceftazidime-avibactam were instead determined from clinical trials and modeled predictions for patients with infections without renal impairment (21, 22). The half-lives of ceftazidime and avibactam were 3.5 and 3.0 h, respectively, and both meropenem and vaborbactam half-lives were 2.0 h in our models. These targeted free drug concentrations and times were extrapolated to simulate humanized concentrations observed in infected patients without renal impairment using PK equations that utilized available data. The same target concentrations were used for each agent for both bolus and extended-infusion administration. The percentage of protein binding for ceftazidime and avibactam was assumed to be 8% for both agents, 2% for meropenem, and 33% for vaborbactam, as shown in Table 2 (23–25).

**TABLE 2:** Values of achieved and targeted serum pharmacokinetic parameters^*a*^

Regimen	fC_max_ (μg/mL)		Half-life (h)		% Protein binding
	Targeted	Achieved	Targeted	Achieved	
Meropenem	26.6	25.0	2.0	2.3	2
Vaborbactam	23.3	25.2	2.0	2.6	33
Ceftazidime	33.8	37.2	3.5	3.8	8
Avibactam	4.8	9.7	3.0	3.8	8

^*a*^ All pharmacokinetic parameters were extrapolated from concentrations taken using bolus administration.

### Susceptibility testing

MICs were confirmed and determined in duplicate using methods described in documents published by the CLSI using CAMHB (26).

### *In vitro* pharmacodynamic model

An *in vitro* 120 h *in vitro* pharmacodynamic (IVPD) model using a 250 mL one-compartment chamber with ports for removal of medium, administration of antimicrobials, and collection of bacterial samples was employed using previously established methodology (27–29). Methods were modified for isolate AR-1046, as it was determined to be a strong fermenter through triple sugar iron tests per the manufacturer’s instructions (Remel R064850). This gas production within the sealed compartment resulted in reduced compartment media volume. To accommodate for this and ensure uniform volume throughout the experiment, models were not vacuum sealed to allow for gas escape. For all treatments simulated against AR-1046, media flow was controlled through the inflow pump, as per the previous methodology, along with an outflow pump, each set to target the respective antibiotic half-life (30). The chamber was prefilled with CAMHB before inoculation with 10^6^ log_10_ colony-forming units (CFU)/mL. Simulated regimens for each isolate included meropenem-vaborbactam (4 g every 8 h), meropenem (2 g every 8 h), ceftazidime-avibactam (2.5 g every 8 h), and antibiotic-free growth controls for comparison. Antibiotic doses were administered via the injection port as either a bolus (administered over ~1 minute) or as an extended infusion (administered over 2–3 h). Before each experiment, *E. cloacae* colonies from an overnight growth on Mueller-Hinton agar (MHA) were used to make an initial starting model inoculum of 10^6^ log_10_CFU/mL. Models were run at least in duplicate and placed in a 35°C water bath with a magnetic stir bar for continuous mixing of media for the duration of the experiment. A third confirmatory run was performed for each regimen if inter-run disagreement was ≥1.0 log_10_ CFU at 24, 48, 72, 96, or 120 h. Samples were taken from models for CFU per milliliter counts at 0, 4, 6, 8, 24, 32, 48, 72, 96, and 120 h (5 days).

### PD analysis

Approximately 1 mL of samples was collected from each model at 0, 4, 6, 8, 24, 32, 48, 72, 96, and 120 h and serially diluted with 0.9% sodium chloride. Bacterial counts were determined by inoculating three 20 µL drops onto MHA plates. Plated samples were incubated at 35°C for 24 h, and colonies were subsequently counted (CFU per mL) with a limit of detection of 2.0 log_10_ CFU/mL (29, 31, 32). Growth curves were conducted for each pathogen at the fastest and slowest half-life of each model. Time-kill curves of colony count (log_10_ CFU/mL) vs time were plotted for each model using SigmaPlot v.13.0 (Systat Software, Inc.). Bactericidal activity (i.e., 99.9% kill) was defined as ≥3 log_10_ CFU/mL reduction in colony counts compared to the initial inoculum, while bacteriostatic activity was defined as a <3 log_10_ CFU/mL reduction in colony count compared to the initial inoculum (33). All PD modeling for target attainment was conducted using one-compartment, first-order elimination models simulating free drug concentration-time profiles.

### PK analysis

Samples of each drug were sent for PK analyses after being obtained through the injection port at 0.5, 4.0, and 8.0 h for verification of target antibiotic concentrations. All samples were stored at −80°C until analysis. High-performance liquid chromatography and liquid chromatography-mass spectrometry methods are described in the Supplemental material.

### Resistance development

Samples of approximately 100 µL were taken from each model at 0, 24, 48, 72, 96, and 120 h and then plated on MHA for MIC determination via the *E*-test for each antimicrobial agent. Manufacture guidelines were followed for all *E*-tests. Any samples with heterogeneous colonies or an MIC shift of two dilutions or more were stored and analyzed further using the standard CLSI MIC method.

### Statistical analysis

Bacterial colony counts were compared at all time points using two-way analysis of variance (ANOVA), followed by Tukey’s post hoc test. Additionally, serum target treatments were compared using a three-way analysis, which included differences in antibiotic administration time. A *P* value of ≤0.05 was considered significant. Data were plotted and graphed using SigmaPlot v.13.0 software (Systat Software, Inc.). All data were analyzed using GraphPad Prism statistical software ( v.10.0.0; GraphPad Prism Inc., Boston, MA).

## RESULTS

### Susceptibility testing

Susceptibility results for both *E. cloacae* isolates are reported in Table 1.

#### IVPD models

**Fig 1:**
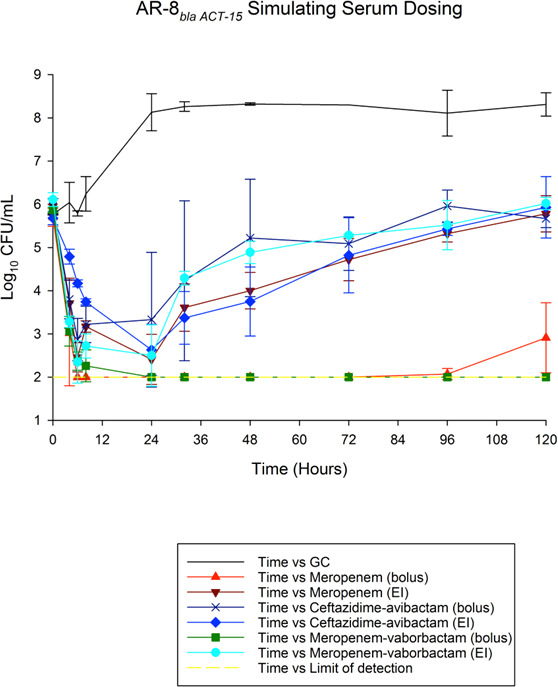
Activities of tested therapies against non-carbapenemase-producing *Enterobacter cloacae*.

**Fig 2:**
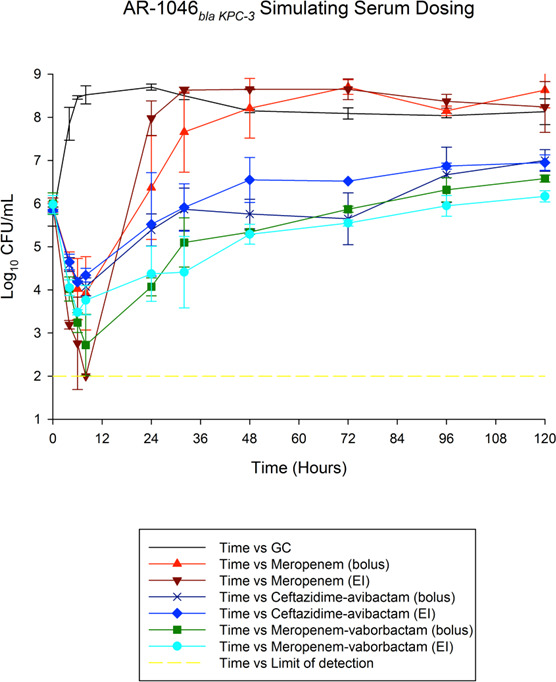
Activities of tested therapies against carbapenemase-producing *Enterobacter cloacae*.

Results for treatment of non-CP and CP CRE strains in 120 h *in vitro* PD models are shown in Fig. 1 and 2, respectively. The achieved PK/PD parameters with either bolus or extended-infusion dosing are displayed in Table 3. Quantitative changes in the bacterial population, expressed as a change in log_10_ CFU/mL, for each antimicrobial regimen are described in Tables 4 and 5 for non-CP *E. cloacae* and CP *E. cloacae*.

**TABLE 3:** Targeted vs achieved serum pharmacokinetic parameters and PK/PD target attainment for β-lactam/β-lactamase inhibitors

Parameter	Isolate	Regimen	Target	Achieved
fT > meropenem MIC	AR-8	Bolus	70%	100%
		EI	90%	99%
	AR-1046	Bolus	45%	47%
		EI	62%	64%
Vaborbactam fAUC 0–24 (mg·h/L)	AR-8 and AR1046	Bolus	604.1 (fAUC/MIC = 1,208.3)	167.5 (fAUC/MIC = 335.0)
		EI	573.6 (fAUC/MIC = 1,147.2)	685.7 (fAUC/MIC = 1,371.3)
fT > ceftazidime-avibactam MIC	AR-8	Bolus	100%	100%
		EI	99%	99%
	AR-1046	Bolus	100%	100%
		EI	99%	99%
Avibactam % fT > CT (1 mg/L)	AR-8 and AR1046	Bolus	100%	100%
		EI	99%	99%

**TABLE 4:** AR-8 *bla_ACT-15_ in vitro* pharmacodynamic model change in bacterial density from initial inoculum (log_10_ CFU/mL) for serum concentrations

Regimen	24 h	48 h	72 h	96 h	120 h
Growth control	+2.26 ± 0.43	+2.45 ± 0.03	+2.43 ± 0.00	+2.24 ± 0.53	+2.44 ± 0.26
Bolus					
Meropenem	−3.7 ± 0.00^*a**,**b*^	−3.7 ± 0.00^*a**,*^^*b*^	−3.7 ± 0.00^*a*^^*,*^^*b*^	−3.63 ± 0.13^*a*^^*,*^^*b*^	−2.79 ± 0.81^*a*^^*,*^^*b*^
Meropenem-vaborbactam	−3.87 ± 0.00^*a**,**b*^	−3.87 ± 0.00^*a*^^*,*^^*b*^	−3.87 ± 0.00^*a*^^*,*^^*b*^	−3.87 ± 0.00^*a*^^*,*^^*b*^	−3.87 ± 0.00^*a*^^*,*^^*b*^
Ceftazidime-avibactam	−2.62 ± 1.56^*a*^^*,**b*^	−0.6 ± 1.36^*a*^	−0.86 ± 0.62^*a*^	+0.01 ± 0.37^*a*^	−0.28 ± 0.21^*a*^
Extended infusion					
Meropenem	−3.54 ± 0.58^*a*^^*,*^^*b*^	−1.95 ± 0.42^*a*^^*,*^^*b*^	−1.23 ± 0.49^*a*^	−0.63 ± 0.19	−0.17 ± 0.42
Meropenem-vaborbactam	−3.61 ± 0.71^*a*^^*,*^^*b*^	−1.23 ± 0.26^*a*^	−0.83 ± 0.03^*a*^	−0.59 ± 0.57^*a*^	−0.09 ± 0.14^*a*^
Ceftazidime-avibactam	−3.06 ± 0.59^*a**,**b*^	−1.93 ± 0.8^*a*^^*,^b^*^	−0.86 ± 0.86^*a*^	−0.25 ± 0.15^*a*^	+0.25 ± 0.71^*a*^

^*a*^ Significantly different compared to growth control.

^*b*^ Significantly different compared to initial inoculum.

**TABLE 5:** AR-1046 *bla_KPC-3_ in vitro* pharmacodynamic model change in bacterial density from initial inoculum (log_10_ CFU/mL) for serum concentrations

Regimen	24 h	48 h	72 h	96 h	120 h
Growth control	+2.78 ± 0.07	+2.23 ± 0.04	+2.17 ± 0.13	+2.12 ± 0.05	+2.21 ± 0.30
Bolus					
Meropenem	+0.46 ± 1.2^*a*^	+2.3 ± 0.69^*b*^	+2.79 ± 0.17^*b*^	+2.24 ± 0.11^*b*^	+2.72 ± 0.41^*b*^
Meropenem-vaborbactam	−1.93 ± 0.21^*a*^^,^^*b*^	−0.67 ± 0.05^*a*^	−0.13 ± 0.07^*a*^	+0.32 ± 0.28^*a*^	+0.58 ± 0.08^*a*^
Ceftazidime-avibactam	−0.54 ± 0.37^*a*^	−0.16 ± 0.34^*a*^	−0.28 ± 0.6^*a*^	+0.75 ± 0.64^*a*^	+1.08 ± 0.25^*a*^
Extended infusion					
Meropenem	+2.04 ± 0.4^*b*^	+2.7 ± 0.01^*b*^	+2.71 ± 0.24^*b*^	+2.43 ± 0.16^*b*^	+2.29 ± 0.59^*b*^
Meropenem-vaborbactam	−1.61 ± 0.64^*a*^^,^^*b*^	−0.69 ± 0.23^*a*^	−0.43 ± 0.07^*a*^	−0.03 ± 0.24^*a*^	+0.19 ± 0.13^*a*^
Ceftazidime-avibactam	−0.32 ± 1.2^*a*^	+0.71 ± 0.52^*a*^	+0.68 ± 0.00^*a*^	+1.03 ± 0.07^*a*^	+1.11 ± 0.18^*a*^

^*a*^ Significantly different compared to growth control.

^*b*^ Significantly different compared to initial inoculum.

#### AR-8: non-CP *Enterobacter cloacae* (*bla_ACT-15_*)

The average bacterial density of the starting inoculum was 5.87 ± 0.18 standard deviation (SD) log_10_ CFU/mL. Growth control models initially grew to 8.13 log_10_ CFU/mL, followed by a period of colony count stabilization, ranging from 8.11 to 8.32 log_10_ CFU/mL, over 120 h. For serum target concentrations, all treatments exhibit bactericidal activity through 24 h, except for the ceftazidime-avibactam bolus treatment, as demonstrated in Table 4. Bacterial regrowth then followed for each of the extended-infusion regimens. Among serum-targeted therapies, meropenem-vaborbactam bolus treatment maintained bactericidal activity for 120 h and did not significantly differ from bolus meropenem, despite observed regrowth with meropenem bolus dosing at 120 h (*P* = 0.39). Interestingly, analysis of meropenem and meropenem-vaborbactam serum targeted treatment using three-way ANOVA revealed that bolus administration was significantly associated with greater antimicrobial activity relative to each agent administered as an extended infusion (*P* < 0.001). Both meropenem and meropenem-vaborbactam showed significantly greater reductions in bacterial colony count compared to ceftazidime-avibactam treatments from 48 to 120 h (*P* < 0.05). While not statistically significant (*P* = 0.08), a difference was also observed between administration methods for ceftazidime-avibactam, with greater antibacterial activity noted within the first 48 h of extended-infusion treatment compared to bolus dosing.

#### AR-1046: CP *Enterobacter cloacae* (*bla_KPC-3_*)

The average bacterial density of the starting inoculum was 5.92 ± 0.16 SD log_10_ CFU/mL. Growth control models initially grew more rapidly and had a greater colony count peak than AR-8, increasing to 8.7 log_10_ CFU/mL after 24 h. There was a slight decline in bacterial colony count after the first 24 h in growth control models, which ranged from 8.04 to 8.7 log_10_ CFU/mL over the 120 h duration. For serum target concentrations, extended-infusion meropenem and bolus meropenem-vaborbactam treatments were associated with bactericidal activity at 8 h. After 24 h, all ceftazidime-avibactam and meropenem-vaborbactam treatments resulted in a bacteriostatic reduction (Table 5). Bacterial regrowth surpassed the initial starting inoculum in all the tested antimicrobials, with the exception of extended-infusion meropenem-vaborbactam, which occurred the latest. Extended-infusion meropenem-vaborbactam was significantly more active than extended-infusion ceftazidime-avibactam at 24 h (*P* = 0.03) and 48 h (*P* = 0.02). In comparison, bolus meropenem-vaborbactam was only significantly different from bolus ceftazidime-avibactam at the 24 h time point (*P* = 0.01). Unlike AR-8 (*bla_ACT-15_*), the duration of antibiotic administration did not result in a significant difference in bactericidal activity for each respective agent, as determined by analysis of meropenem, meropenem-vaborbactam, and ceftazidime-avibactam serum concentration-targeted treatments (*P* > 0.1 for all treatments) using three-way ANOVA.

### Development of resistance

#### AR-8: non-CP *Enterobacter cloacae* (*bla_ACT_*_*-15*_)

Newly acquired antimicrobial resistance occurred during treatment with extended-infusion ceftazidime-avibactam, resulting in MIC shifts from 1 to 4 μg/mL after 72 h. Extended-infusion meropenem resulted in shifts from 2 to >32 µg/mL after 120 h, while no MIC shifts were observed from the meropenem-vaborbactam treatment.

#### AR-1046: CP *Enterobacter cloacae* (*bla_KPC_*_*-3*_)

Similarly, resistance emergence was observed in models of meropenem and ceftazidime-avibactam. MICs shifted from 8 to >32 µg/mL in serum meropenem models after 24 h of antimicrobial exposure. Ceftazidime-avibactam serum models exhibited MIC shifts from 2 to 16 μg/mL after 120 h of treatment. No MIC shifts were observed with serum meropenem-vaborbactam.

## DISCUSSION

CREs are classified as an urgent threat by the CDC, with *Enterobacter cloacae* being the most prevalent organism designated as non-CP CRE (9, 34). This resistance can significantly limit therapeutic options and lead to the use of more toxic or less effective treatment (2). This is especially concerning for *Enterobacter cloacae*, which frequently harbors multiple resistance mechanisms that have a combined effect, leading to decreased antibiotic susceptibility and potentially antimicrobial failure, as well as worse patient outcomes (35). This abundance of resistance mechanisms can lead to delays in initiating appropriate therapy and may lead to increased morbidity and mortality (36). Furthermore, the optimal treatment for CRE remains unknown, particularly when MIC distributions are clustered around clinical breakpoints, as is the case with AR-8. Extended-infusion carbapenems are a proposed treatment option for non-CP CREs that remain susceptible to meropenem and/or imipenem, depending on disease severity. Data are limited regarding the effectiveness of extended-infusion meropenem monotherapy for treating non-CP CRE, especially when compared to newer BLBLI combinations, such as meropenem-vaborbactam (14). The addition of β-lactamase inhibitors like vaborbactam has a theoretical benefit of overcoming β-lactamase overexpression, coinciding with reduced MICs (14). Therefore, our study aimed to evaluate the optimal therapy against CP and non-CP CRE isolates by examining the concentrations achieved in patients receiving standardized doses.

This work identifies several important considerations for antimicrobial selection in patients infected with both CP and non-CP CRE. First, treatment with all three antimicrobials resulted in bactericidal activity at 24 h for the non-CP isolate, followed by regrowth in nearly all regimens. The only treatment that did not result in bacterial regrowth through 120 h was meropenem-vaborbactam administered as a bolus. These results did not differ significantly from those of meropenem bolus dosing (*P* = 0.39); however, some bacterial regrowth was observed at 120 h in the meropenem bolus group for this isolate. Regardless, these results do support the IDSA guidance recommendations to consider still utilizing meropenem for non-CP CRE from an efficacy perspective. However, our results indicate that EI carbapenem treatment may be suboptimal compared to BLBLI treatment with respect to resistance development for isolates with MICs around the clinical breakpoints, like AR-8. The PD index most closely associated with β-lactam activity is time dependent, with a treatment goal of maintaining free drug concentrations above the MIC for a high percentage of the time (fT >MIC). Because of this, extended-infusion administration has emerged as a method to increase the time above MIC (37). PK/PD target attainment studies have indicated that for *Enterobacterales*, meropenem target concentrations should achieve an fT >MIC of 40%–45% and for ceftazidime, the minimum goal fT >MIC should be at least 50%, which was achieved in our models (99%–100% fT >MIC for ceftazidime-avibactam treatments) (21, 38). There are several different PD targets proposed to evaluate avibactam activity, and they vary depending on the organism and type of model. The most frequently reported target is the time fraction of free drug above a threshold concentration (fT >C_T_), with C_T_ typically ranging from 0.5 to 1.0 mg/L for *Enterobacterales* and more specifically *E. cloacae* (39, 40). This differs slightly for single-compartment *in vitro* models, which instead have shown area under the curve to be better correlated with activity; however, avibactam concentrations of >1.0 mg/L have similarly been shown to produce maximal bacterial clearance for *E. cloacae* (41). This was observed throughout the entirety of the bolus models and for 99% of the time with EI. Our study interestingly showed that bolus administration of meropenem-containing regimens led to the highest reduction in bacterial counts in the non-CP strain with multiple porin mutations despite adequate target attainment with EI administration. Resistance development, presumed through MIC shifts, was not frequently observed in serum target models for this isolate and was only noted for extended-infusion meropenem and extended-infusion ceftazidime-avibactam, which may indicate a protective effect of vaborbactam in minimizing MIC shifts. Bacterial regrowth, despite the absence of any associated MIC shifts, could represent the *in vitro* phenomenon of bacterial tolerance; however, this cannot be confirmed from the results obtained in the present study (42). Furthermore, previous studies have demonstrated that porin mutations present in *Enterobacterales* have a greater impact on carbapenem activity than ceftazidime-avibactam and that greater meropenem-induced bacterial stress reduces porin expression (43, 44). This suggests that there may be a benefit from initial high bolus doses to achieve rapid therapeutic concentrations or that subtherapeutic periods reduced antibiotic stress for isolates with multiple porin mutations, although further studies are warranted.

CP *Enterobacterales* remain challenging to treat due to the presence of one of these enzymes, which confers resistance to most β-lactams, including variable *in vitro* activity of carbapenems (45). Among CRE isolates in the United States, 35% possess at least one carbapenemase gene, which is even more common (83% of CRE isolates are carbapenemase producing) when defined as resistance to meropenem or imipenem (7, 8). Mutations in carbapenemases have been identified following the introduction and increased use of novel BLBLI combination antibiotics, which impact antimicrobial susceptibility. One such mutation is *bla*_*KPC-3*_, which exhibits greater hydrolytic activity against ceftazidime-avibactam than its *bla*_KPC-2_ counterpart (46). Furthermore, enzymatic mutations at the D179 position, which lead to elevated ceftazidime-avibactam MICs in tandem with reduced meropenem MICs, have been reported (47). The current literature cited in the 2024 IDSA AMR Gram-Negative Guidance document estimates the emergence of resistance to ceftazidime-avibactam and meropenem-vaborbactam following treatment of CRE to be 10% and 3%, respectively (14, 17, 48–54). Our study demonstrated that resistance emergence, particularly with the *bla*_*KPC-3*_ isolate, was more prevalent with ceftazidime-avibactam treatment compared to meropenem-vaborbactam. The lack of MIC shifts following meropenem-vaborbactam treatment in serum-targeted models is supported by previous *in vitro* studies, which showed that a simulated dosage regimen of 2 g meropenem and 2 g vaborbactam suppressed the development of resistance (55).

Our study has several limitations. This is an IVPD model with a limited duration of 120 h, which does not consider a patient’s own immune function, thereby limiting its extrapolation to clinical outcomes. Additionally, this study simulated only two strains of *Enterobacter cloacae* at a fixed initial inoculum of 10^6^ CFU/mL, and final observations may not predict the results of a higher burden infection. It evaluated only three antimicrobial agents and did not account for moderate to severe renal impairment. The antibiotic concentrations targeted in our models were selected to simulate infected patients and were notably lower than those listed in each compound’s respective USP. This is consistent with lower concentrations observed in patients compared to those obtained from healthy human subjects; however, this should be taken into consideration when extrapolating these results (22–24). Therefore, it is unclear whether other antimicrobial agents provide more optimal reductions in bacterial counts and how varying degrees of renal function might alter PD effects against these isolates, as this can affect drug accumulation, potentially leading to higher exposures. However, this effect can be mitigated by adjusting the renal dosage (56). Additional drug concentration sampling for extended-infusion models was not performed, which may potentially bias PK/PD extrapolation from these results. Nonetheless, this study provides critical insight into the difficulties of eradicating carbapenem-resistant *E. cloacae*. Both CP and non-CP isolates demonstrate a propensity for inducible resistance mechanisms, posing a serious threat to public health. Data indicate there is an unmet clinical need to identify optimal therapy for CRE to improve patient outcomes.

### Conclusions

Meropenem-vaborbactam humanized serum concentrations demonstrated the highest activity against CP-CRE *E. cloacae* compared to meropenem and ceftazidime-avibactam in IVPD models. For the non-CP isolate, the most active treatments were meropenem-vaborbactam and meropenem, which showed similar activity. Resistance development, as represented by MIC shifts, was observed only with meropenem and was not observed with meropenem-vaborbactam. Our results should be applied to clinical practice cautiously, as confirmation from clinical outcome trials is necessary.

## SUPPLEMENTAL MATERIAL

Supplemental material aac.00906-25-s0001.docx Table S1.

ASM does not own the copyrights to Supplemental Material that may be linked to, or accessed through, an article. The authors have granted ASM a non-exclusive, world-wide license to publish the Supplemental Material files. Please contact the corresponding author directly for reuse.
