# Supplementary material for: Unlocking potentials: the impact of meropenem, meropenem-vaborbactam, and ceftazidime-avibactam in combating carbapenem-resistant Enterobacter cloacae
Source: Antimicrob Agents Chemother. 2025 Nov 19;69(12):e00906-25. doi: 10.1128/aac.00906-25 (PMC12691640; doi:10.1128/aac.00906-25)
Supplement: Supplemental material — Table S1. [file aac.00906-25-s0001.docx]

**Supplementary Appendix**

**HPLC analysis of Meropenem**

Meropenem samples were prepared and analyzed as previously outlined with modifications.[59] Meropenem calibration curve standards and quality controls were prepared in 21 mg/mL MHB at nominal concentrations of 0.25, 1, 2, 3, 5, 20, 30, and 50 μg/mL for standards, and 0.5, 10, and 40 μg/mL for quality controls. Acetaminophen was used as the internal standard at 25 μg/mL in methanol. All solutions were freshly prepared on the day of analysis. Aliquots of 200 μL serum samples, calibration standards, and quality controls were spiked with 50 μL of internal standard, mixed thoroughly, and centrifuged at 1,500 RCF for 5 minutes at room temperature. The supernatants were transferred to 2 mL HPLC vials.

A 60 μL injection of meropenem samples, stored in the autosampler at room temperature, was separated using an Ultimate 3000 HPLC system (Thermo Scientific, Waltham, MA, USA) equipped with a Hypersil Phenyl-BDS C18 column (4.6 x 150 mm, 5 μm; Thermo Scientific, Waltham, MA, USA) and a µBondapak C18 guard column (125Å, 10 μm, Waters Corporation, Milford, MA, USA) maintained at room temperature. Chromatographic separation was achieved using an isocratic gradient with a mobile phase consisting of 25 mM sodium phosphate buffer (pH 6.5) and 13% methanol. The total run time was 20 minutes, at a flow rate of 1.0 mL/min. Detection was performed using a programmable UV detector set at 240 nm for acetaminophen and 298 nm for meropenem. Chromeleon version 7.2.9.11323 software (Thermo Scientific, Waltham, MA, USA) was used for quantification, employing an average calibration curve generated from two curves per batch. Linear regression based on the peak area ratio (analyte/internal standard) against nominal concentration was used without weighting.

**LC-MS/MS analysis of Ceftazidime-Avibactam and Vaborbactam**

***Ceftazidime-Avibactam sample preparation.*** Ceftazidime-avibactam, co-formulated at a 4:1 ratio, calibration curve standards and quality controls were prepared in 21 mg/mL MHB at nominal concentrations of 0.25, 1, 2, 3, 5, 10, 20, 30, and 50 μg/mL for standards, and 0.5, 1.5, 10, 15, and 40 μg/mL for quality controls. Acetaminophen was used as the internal standard at 0.25 μg/mL in acetonitrile. All solutions were freshly prepared on the day of analysis. Aliquots of 100 μL serum samples, calibration standards, and quality controls were spiked with 200 μL of internal standard, mixed thoroughly, and centrifuged at 1,500 RCF for 5 minutes at 4°C. The supernatants were transferred to 2 mL HPLC vials for LC-MS/MS analysis.

***Vaborbactam sample preparation.*** Vaborbactam calibration curve standards and quality controls were prepared in 21 mg/mL MHB at nominal concentrations of 1, 2, 3, 5, 10, 20, 30, and 50 μg/mL for standards, and 1.5, 15, and 40 μg/mL for quality controls. Phenacetin was used as the internal standard at 0.5 μg/mL in acetonitrile. All solutions were freshly prepared on the day of analysis. Aliquots of 100 μL serum samples, calibration standards, and quality controls were spiked with 100 μL of internal standard, mixed thoroughly, and centrifuged at 1,500 RCF for 5 minutes at 4°C. The supernatants were transferred to 2 mL HPLC vials for LC-MS/MS analysis.

***LC-MS/MS conditions.*** A 5-μL injection of ceftazidime-avibactam samples, stored in the autosampler at 4°C, was separated using a 1290 Infinity II UPLC system (Agilent, Santa Clara, CA, USA) equipped with a Hypersil Gold C18 column (2.1 × 100 mm, 1.9 μm; Thermo Scientific, Waltham, MA, USA) and an Acquity BEH C18 guard column (2.1 x 5 mm, 1.7 um; Waters Corporation, Milford, MA, USA) maintained at 40°C. Chromatographic separation was performed using a linear gradient with mobile phase A, consisting of 1 g/L ammonium formate in water with 0.1% formic acid, and mobile phase B, containing 0.1% formic acid in acetonitrile. The total run time was 7 minutes at a flow rate of 0.25 mL/min. The gradient program began with 0% mobile phase B from 0 to 0.2 minutes, followed by a gradual increase from 0% to 100% mobile phase B between 0.21 and 4 minutes. The composition was maintained at 100% mobile phase B from 4.01 to 5 minutes before transitioning back to 0% between 5.01 and 5.1 minutes. Finally, the system was held at 0% mobile phase B from 5.11 to 7 minutes.

A 5-μL injection of vaborbactam samples, stored in the autosampler at 4°C, was analyzed using the same UPLC system, equipped with an Acquity BEH C18 column (2.1 × 50 mm, 1.7 μm; Waters Corporation, Milford, MA, USA) and an Acquity BEH C18 guard column (2.1 x 5 mm, 1.7 um; Waters Corporation, Milford, MA, USA) maintained at 40°C. Chromatographic separation was performed using a linear gradient with mobile phase A, consisting of 0.1% formic acid in water, and mobile phase B, containing 0.1% formic acid in acetonitrile. The total run time was 5 minutes at a flow rate of 0.5 mL/min. The gradient program began with 0% mobile phase B from 0 to 0.2 minutes, followed by a gradual increase from 0% to 100% mobile phase B between 0.21 and 3 minutes. The composition was maintained at 100% mobile phase B from 3.01 to 4 minutes before transitioning back to 0% between 4.01 and 4.1 minutes. Finally, the system was held at 0% mobile phase B from 4.11 to 5 minutes.

Detection was performed using an Agilent 6470 LC-triple quadrupole mass spectrometer operated in positive and negative electrospray ionization mode with dynamic multiple-reaction monitoring. Source parameters included gas temperature (325°C), gas flow (13 L/min), sheath gas temperature (375°C), sheath gas flow (11 L/min), nebulizer pressure (40 psi), capillary voltage (2500 V), and nozzle voltage (500 V). Mass spectrometry parameters are listed in **Table S1.**

Agilent MassHunter version 10.1 software was used for quantification, employing an average calibration curve generated from three curves per batch. Linear regression based on the peak area ratio (analyte/internal standard) against nominal concentration was used without weighting and with a forced origin.

| **Supplementary Table 1. Mass spectrometry parameters** | | | | | | | |
| --- | --- | --- | --- | --- | --- | --- | --- |
| **Compound** | **Precursor ion (m/z)** | **Product ion (m/z)** | **Dwell time (ms)** | **Fragmentor (V)** | **Collision energy (V)** | **Cell accelerator (V)** | **Polarity** |
| Ceftazidime | 547.1 | 468 | 200 | 74 | 18 | 4 | Positive |
| Acetaminophen | 152.1 | 110 | 200 | 116 | 17 | 4 | Positive |
| Avibactam | 264 | 96 | 200 | 128 | 33 | 4 | Negative |
| Vaborbactam | 298.1 | 220.1 | 200 | 68 | 29 | 4 | Positive |
| Phenacetin | 180.1 | 110 | 200 | 134 | 25 | 4 | Positive |

REFERENCE:

59. Sutherland CA, Nicolau DP. 2020. Development of an HPLC method for the determination of meropenem/vaborbactam in biological and aqueous ma Method for the Determination of Meropenem/Vaborbactam in Biological and Aqueous Matrixes. J Chromatogr Sci 58:726–730. https://doi.org/10.1093/chromsci/bmaa041
